# Decoding the Contribution of IAPP Amyloid Aggregation to Beta Cell Dysfunction: A Systematic Review and Epistemic Meta-Analysis of Type 1 Diabetes

**DOI:** 10.3390/ijms26020767

**Published:** 2025-01-17

**Authors:** Valeria Moya-Gudiño, Nelly F. Altamirano-Bustamante, Cristina Revilla-Monsalve, Myriam M. Altamirano-Bustamante

**Affiliations:** 1 Unidad de Investigación en Enfermedades Metabólicas, Centro Médico Nacional Siglo XXI, Instituto Mexicano del Seguro Social, Mexico City 06720, Mexico; moguval1704@gmail.com (V.M.-G.); cristina_revilla@hotmail.com (C.R.-M.); 2 Servicio de Endocrinología, Instituto Nacional de Pediatría, Mexico City 04530, Mexico

**Keywords:** diabetes mellitus type 1, DM1, IAPP, amyloid aggregation, apoptosis, pancreatic islets, autoimmune, oligomers

## Abstract

Diabetes Mellitus Type 1 (DM1) is an autoimmune disease characterized by the destruction of beta cells in the pancreas. Although amyloid formation has been well-studied in Diabetes Mellitus Type 2 (DM2), its role in DM1 remains unclear. Understanding how islet amyloid polypeptide (IAPP) contributes to beta cell dysfunction and death in DM1 could provide critical insights into disease mechanisms and pave the way for novel diagnostic and therapeutic strategies. A systematic review and epistemic meta-analysis was conducted using a modified PICO framework, focusing on studies related to DM1 and the IAPP aggregation process. Searches in PubMed, BIREME, and Web of Science yielded 37 relevant articles, which were analyzed and individually evaluated based on specific quality criteria. Studies that experimentally identified the formation of IAPP oligomers in DM1 were selected, along with relevant review articles. Experimental studies from human and animal models detected the presence of IAPP oligomers in DM1 patients, as well as in nonobese diabetic (NOD) and homozygous mice. Techniques like Western Blot (WB), Transmission Electron Microscopy (TEM) and Congo red staining detected various oligomers sizes, with smaller ones showing higher cytotoxicity. IAPP oligomers have been detected in the pancreatic islets of DM1 patients, contributing to beta cell damage and disease progression.

## 1. Introduction

The role of IAPP aggregation in Diabetes Mellitus Type 2 (DM2) is well-established, but its implications in Diabetes Mellitus Type 1 (DM1) remain enigmatic. Unraveling the role of amyloid peptides, particularly IAPP, in the pathogenesis of DM1 has become a key focus in scientific research. IAPP is well-known for its role in pancreatic beta-cell dysfunction and death, particularly in DM2. However, recent studies have revealed that these aggregations may also appear in patients with DM1, thus raising the question: Are IAPP oligomers formed in DM1? If so, what are their structural and functional characteristics? What effect do these aggregates have on beta cells and the immune system? And what is the role of oligomerization-fibrilization process of IAPP in the pathogenesis of the disease? Therefore, this work seeks to address these questions under the hypothesis that IAPP aggregation and its misfolding products are central to the pathogenesis in DM1. Unlike in DM2, where metabolic stress drives IAPP aggregation, the process in DM1 is linked to the autoimmune destruction of beta cells. Specifically, the formation of toxic IAPP oligomers may exacerbate immune-mediated damage, creating a feedback loop that accelerates beta-cell apoptosis and inflammation.

To address these knowledge gaps, this review aims to characterize the structural and functional properties of IAPP oligomers formed in DM1, identify the mechanism linking IAPP oligomer formation with beta-cell dysfunction, examine the role of the IAPP oligomerization-fibrillization process in immune-mediated beta-cell damage, and evaluate DM1 as a conformational disease to identify novel diagnostic and therapeutic targets.

**Figure 1:**
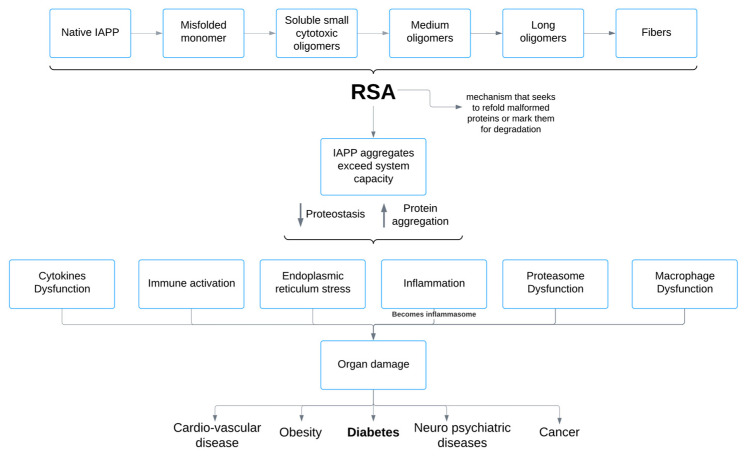
Pathological Pathway of Oligomerization-Fibrilization Process of IAPP and its impact on Diseases. Shown in the diagram first is the process of oligomerization-fibrilization of IAPP starting from its native form and when this process is very active it saturates the RSA starting from its native form and progressing to amyloid fiber formation. RSA is activated in an attempt to counteract misfolding; however, when RSA is insufficient to maintain proteostasis, it leads to immune activation, ER stress, inflammation, macrophage dysfunction, cytokines and proteasome dysfunction. The inflammation becomes inflammasome and this cascade ultimately results in organ damage and contributes to the development of diseases such as diabetes mellitus, cardiovascular disease, obesity, neuropsychiatric disorders, and cancer.

Evidence supporting the hypothesis comes from a variety of human, animal, and cellular studies. Here we report (1) that patients with DM1 exhibit detectable levels of IAPP in their blood, although these findings are varied: Tomita showed lower levels [1], while Paulsson et al. observed that IAPP concentrations were high [2], (2) studies that have demonstrated the presence of amyloid deposits in islet transplants and the detection of soluble IAPP oligomers in DM1 patients [3,4,5], (3) other investigations into the pancreatic islets from DM1 patients that have confirmed IAPP deposits [6,7]. As shown in the roadmap depicting the pathological pathway of the oligomerization-fibrilization process of IAPP in Figure 1.

This review also highlights the identification of IAPP oligomer epitopes that activate the immune response mediated by cytotoxic T cells [8,9]. This aligns with findings from animal models, which report the formation of IAPP oligomers during the early stages of DM1 [10]. Additionally, an accumulation of IAPP-reactive CD4+ T cells in the pancreas have been documented, implicating IAPP aggregation in autoimmune processes [11]. Finally, several review studies point out the importance of IAPP oligomers in beta cell apoptosis in DM1. ER stress is mentioned to contribute significantly to the pathogenesis of the disease. 

## 2. Materials and Methods

The systematic review followed a modified PICO (Participants/Intervention/Comparison/Outcome) framework (PIO). The first step involved was formulating the research question: “Do amyloids form in Diabetes Mellitus Type 1?”. As the study relied on existing literature, no ethical approval or informed consent was required. All articles and data referenced can be accessed from the databases used.

### 2.1. Data Sources and Searches

The databases used were PubMed, BIREME, and Web of Science. The research began on 31 July 2024, following the PIO and PRISMA protocols. It is worth mentioning that the Comparison (C) was excluded from the protocol because the objective of this research is to seek new information despite the research question. Each term of the PIO protocol was searched in each database in the same manner.

P (Participants): Type 1 Diabetes, DM1 and IAPP.

I (Intervention): Amyloid, Amyloidosis, Aggregation, Fiber and Oligomerization.

O (Outcome): Cytotoxicity, Glucotoxicity and Apoptosis.

**Figure 2:**
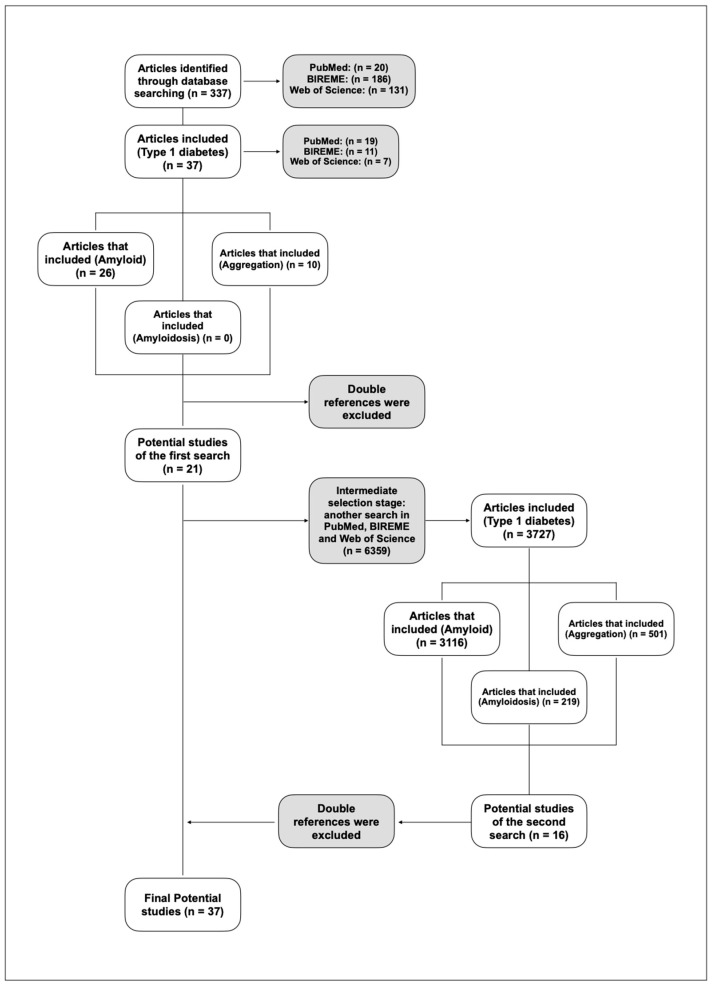
Modified PICO (PIO) for the article selection process. PRISMA Flowchart review process for the analyzed articles.

Search terms were grouped and queried using Boolean operators (‘OR’ and ‘AND’) for comprehensive database exploration. The search algorithm was saved in a RIS file and uploaded to Mendeley Reference Manager. A PRISMA flowchart was made to show the history of the research (Figure 2). The diagram describes the key words that were searched in the articles and the way in which studies were discarded.

A second search was performed because the above-mentioned search did not retrieve a sufficient number of articles relevant to the research question. The search form was as follows: ((Type 1 diabetes) OR (Streptozotocin)) AND ((Amyloid) OR (Oligomers) OR (IAPP)).

### 2.2. Eligibility Criteria

A total of 337 articles were retrieved from the three databases in the first search (20 from PubMed, 186 from BIREME, and 131 from Web of Science). Mendeley was used to select relevant articles, focusing on those related to DM1 while excluding DM2. Subsequently, the articles were checked to ensure they contained the words ‘aggregation’, ‘amyloid’, and ‘amyloidosis’, which were searched independently in each database. This process resulted in 21 potentially relevant articles.

**Figure 3:**
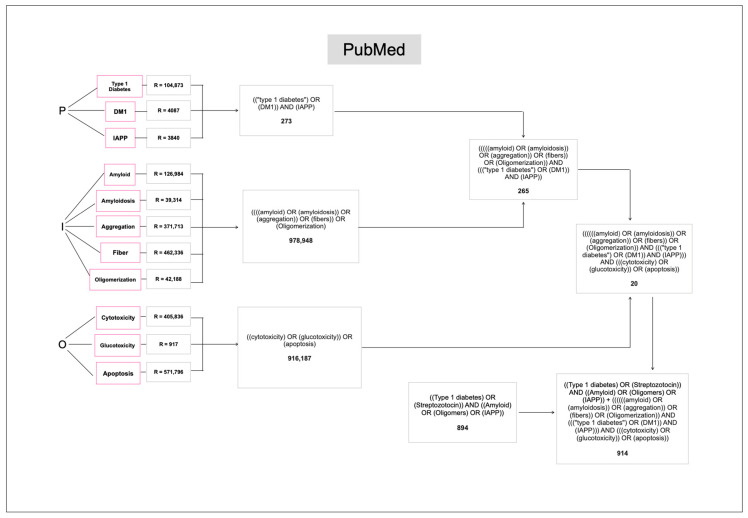
Modified PICO (PIO) approach for the systematic review from the database PubMed. P (Participants), I (Intervention) and O (Outcome).

In the second search, a total of 6359 were obtained from the three databases (894 from PubMed, 2809 from BIREME and 2656 from Web of Science) (Figure 3, Figures S1 and S2). A total of 16 relevant articles were obtained from the research question. Finally, combining the results from both searches yielded a total of 37 potentially relevant articles.

### 2.3. Data Extraction and Quality Assessment

The information was extracted by creating diagrams for each article, focusing on the objective, study population, research question, methods used, and results. The Quality (%) assessment was based on the PRISMA approach, applying the following criteria: (1) a well-defined objective; (2) addressing the research question; (3) clear definition of the concepts that were researched and measured; (4) an extensive and detailed description of the methodology used; (5) description of the measuring instruments (reliable and valid); (6) sample size and characteristics of the target population and the participants; (7) adequate statistical analysis; and (8) well-founded conclusions.

Each criterion was assigned a value of 12.5%, totaling 100%. To make the research more valuable, only articles with a score of 75% or more were considered for a deeper analysis, which are included in Table S1 which included: author, year of publication and setting, target molecule, detection methods, target population, relevant results and written informed consent.

### 2.4. Epistemic Meta-Analysis 

Meta-analysis is a research technique that involves reviewing and integrating existing studies, theories, or findings. The term "epistemic" relates to the investigation of knowledge, focusing on analyzing the fundamental assumptions, research methods, and theoretical frameworks behind oligomerization-fibrillization of IAPP in DM1 and their roles in the DM1 as conformational disease. This approach aims to offer a clearer insight into the current body of knowledge, identifying its strengths, weaknesses, and areas where further research is needed. 

## 3. Results

The review involved a search for articles across three databases: PubMed (*n* = 914), BIREME (*n* = 2995), and Web of Science (*n* = 2787), yielding a total of 6696 articles related to the research question “Do amyloids form in DM1?” using the modified PICO protocol (PIO). Articles that received a Quality score higher than 75% (*n* = 20) were analyzed in greater detail and are described in Tables S1 and S2.

**Figure 4:**
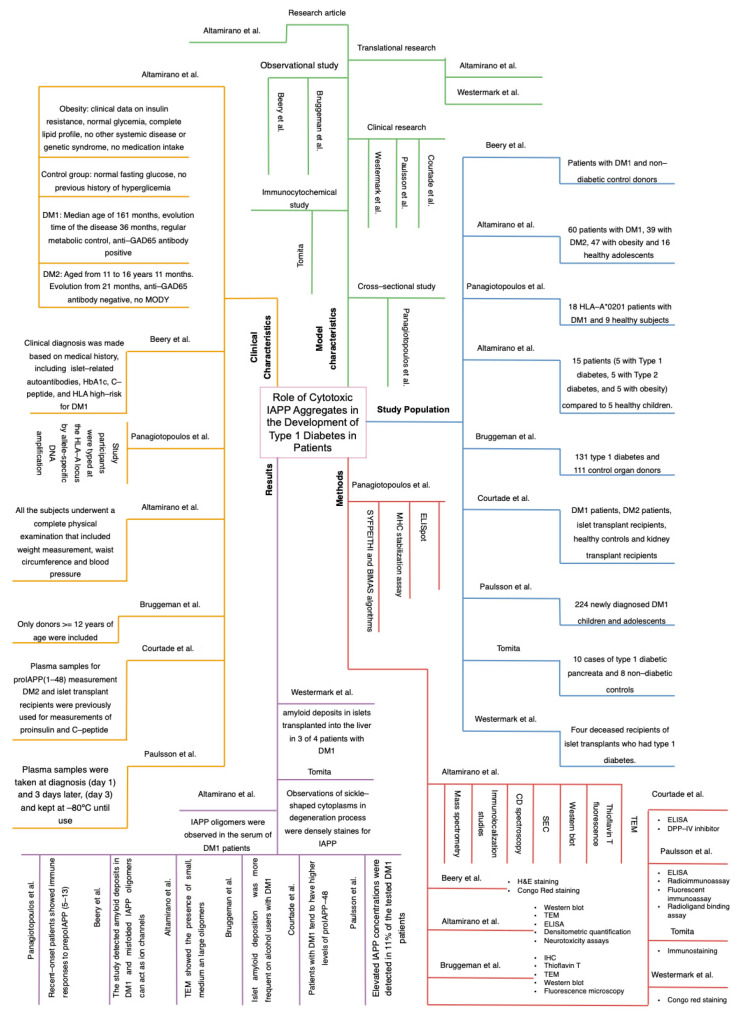
Graphical Summary of Results in Human Models. The impact of Amyloid Formation on Beta Cell Function on patients with DM1 is shown in this figure. It can be seen that in the right of the diagram corresponds to the study population (Blue), the upper right quadrant belongs to the model characteristics (Green), on the upper left side of the diagram are the clinical characteristics (Yellow), on the lower left part corresponds to results (Purple) and finally the lower right part are the methods (Red). This map structure is the same in the three diagrams and allows to have a structured vision of the key information. This figure is based on data and analysis from references [1,2,3,4,5,6,7,8,12].

**Figure 5:**
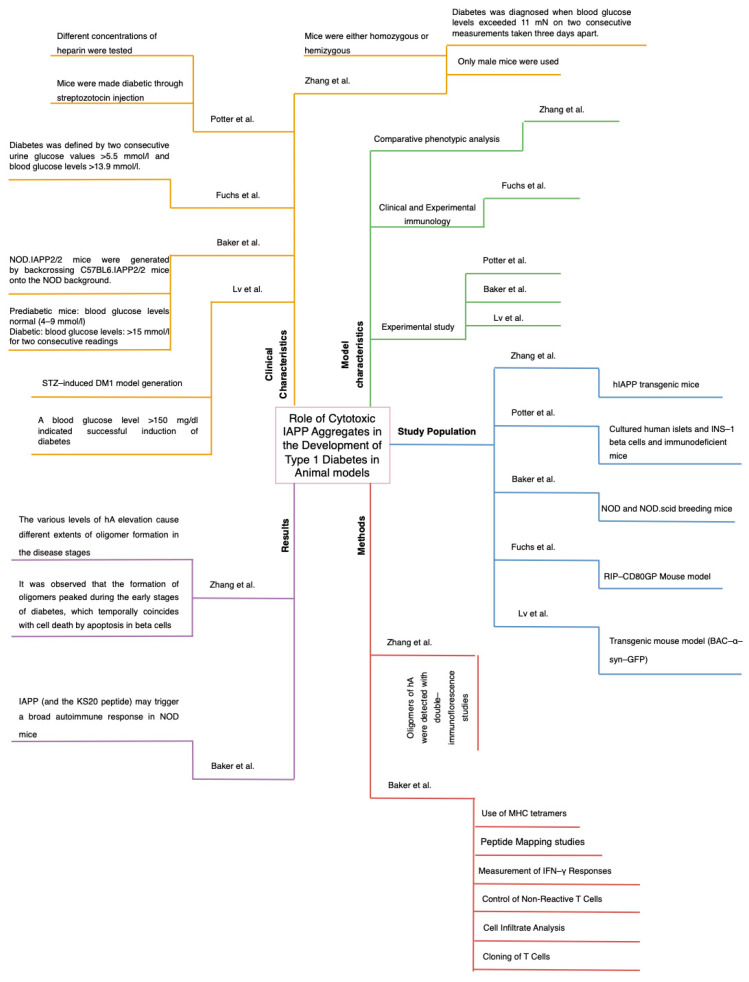
Graphical Summary of Results in Animal Models. In this figure the same structure as that of Figure 4 was applied but focused to animal models. The layout remains consistent with the study population represented in blue on the right side of the diagram, model characteristics in green in the upper right quadrant, clinical characteristics in yellow on the upper left, results in purple on the lower left, and methods in red on the lower right. This standardized mapping allows for a clear comparison between the different models used, highlighting key similarities and differences across both animal and human studies. This figure is based on data and analysis from references [10,11,13,14,15].

**Figure 6:**
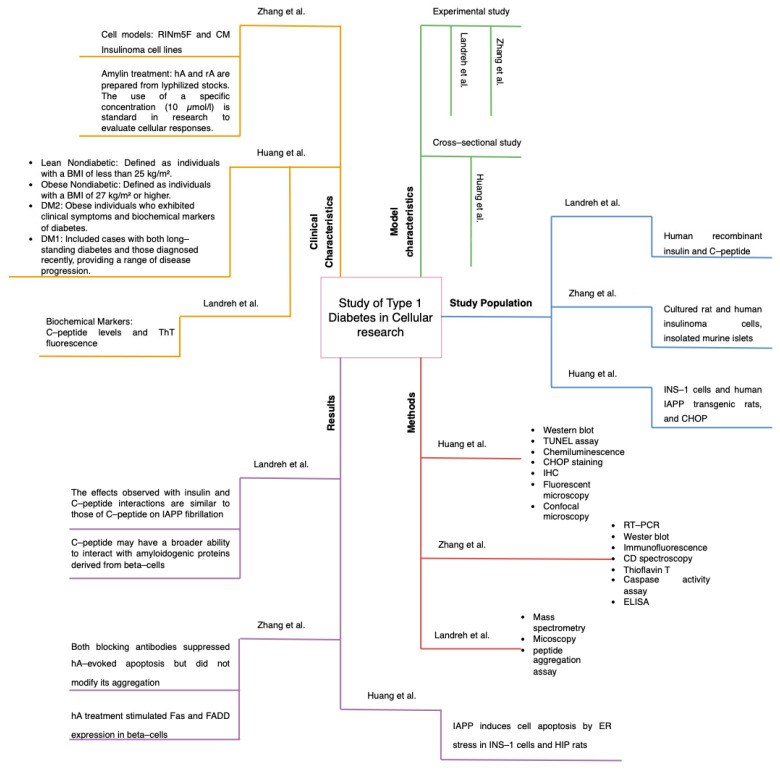
Graphical Summary of Results in Cellular Models. In this figure the same diagrammatic structure used for Figure 4 and Figure 5 was applied, and the organization remains consistent. This framework facilitates a direct comparation between cellular models and both animal and human studies. This figure is based on data and analysis from references [16,17,18].

Three diagrams were also created where it synthesizes the information obtained from the 20 studies (Figure 4, Figure 5 and Figure 6). This figures were separated into animal, cellular and human models and the diagrams were divided into 5 quadrants, which are results, methods, study population, and clinical and model characteristics. The same structure is applied to all three diagrams, helping to quickly compare and analyze the information.

### 3.1. Pathological Pathway of IAPP Aggregation

The aggregation of IAPP is a trigger of various metabolic disorders, particularly diabetes mellitus. Misfolded IAPP monomers aggregate into toxic oligomers and amyloid fibers, provoking the disruption of cellular homeostasis and triggering pathological cascades. A critical cellular response to these aggregates is Refolding System Activation (RSA), which refers to the process of triggering the cellular machinery responsible for restoring the correct structure of misfolded or unfolded proteins. When the RSA response is insufficient to maintain proteostasis, it leads to immune activation, endoplasmic reticulum (ER) stress, inflammation, and proteasome dysfunction. These disruptions cause organ damage, that finally leads to diseases like diabetes, cardiovascular complications, obesity, neuropsychiatric disorders and cancer (Figure 1).

This study explores the crucial role of IAPP aggregation in the pathogenesis of DM1. Previous evidence has demonstrated the presence of IAPP oligomers in the disease, highlighting their contribution to beta-cell dysfunction. Building on this foundation, the results presented here investigate the progression of IAPP aggregation and its broader pathological implications.

### 3.2. Presence of IAPP Oligomers and Amyloid Deposits in DM1: Evidence and Detection Methods

Two studies investigated the presence of natural oligomeric aggregates of IAPP (RIAO) [4,5], one in the blood of 146 pediatric patients with obesity or diabetes, as well as 16 healthy children, and the other on healthy individuals, DM1 and DM2 patients (Table S1). RIAO presence was confirmed through Western Blot (WB) analysis and the oligomers exhibited varying degrees of aggregation (trimers, hexamers and dodecamers). Transmission Electron Microscopy (TEM) showed large, medium and small oligomers in DM1, with indications of oligomers merging and unfolding to form long, wide fibers, as well as small fibers. Cytotoxicity and kinetics was also studied, demonstrating significant toxicity associated with RIAO and rapid aggregation in DM1 (Table S2).

Amyloid deposits in the islets of organ donors has been reported in several studies. Histopathological analyses of hematoxylin and eosin (H&E) and immunocytochemistry (ICC) revealed amyloidosis in the islets of the donors. Beta cells were displaced toward the islet periphery due to intra-islet amyloid deposits [6] and it was observed that amyloid deposits were more frequently in alcohol consumers [7]. However, contrasting findings were reported. A histological analysis was conducted on the pancreases from 12 islet antibody-positive DM1 patients and 19 healthy age-matched controls (Table S1). Using Congo red staining to identify amyloid deposits, the study found no evidence of amyloid deposition in either DM1 or control islets [19]. 

To further understand how altered expression and aggregation of human amylin (hA) contribute to cellular degeneration and the progression of diabetes, a study employed animal models. The phenotypes of homozygous (DM1 model) and hemizygous transgenic hA mice was compared. Using the A11 antibody, immunofluorescence tests were conducted on permeabilized and antigen-retrieved pancreatic tissue sections. Immunoreactive signals corresponding to amylin oligomer-like immunoreactive material (AOLIM) were observed (Table S2). These findings confirmed the formation of intracellular and extracellular IAPP oligomers in pancreatic beta cells at various stages of DM1 [10].

Using a streptozotocin (STZ)-induced DM1 model with transgenic mice overexpressing human α-synuclein, we observed that STZ treatment increased the insoluble fraction of α-synuclein, promoting its aggregation under hyperglycemic conditions. Additionally, enhanced α-synuclein deposition was detected in the pancreatic islets of these mice [15]. While the study primarily investigates α-synuclein aggregation, the observed deposition in pancreatic islets suggests a potential shared pathway influencing protein aggregation, including IAPP. Furthermore, our group demonstrated that rIAPP aggregates into amyloid oligomers (trimers and hexamers) through self-association or hetero-assembly. These findings are the first to show that rIAPP amyloid oligomers contribute to the development of STZ-induced diabetes in rats [20].

### 3.3. Refolding System Activation

Now that the presence of IAPP aggregates in DM1 has been established, its important to understand the consequences of this accumulation of misfolded proteins. The refolding system activation (RSA), which refers to the set of cellular mechanisms that help proteins regain their functional shape after being misfolded due to stress or environmental changes, plays a crucial role in this context.

Due to the activation of the refolding system, several mechanisms are triggered within the cell. In this work we focused on mechanisms such as ER stress, inflammation, proteasome dysfunction and immune activation based on the articles that were obtained from the databases.

### 3.4. Immune Response to IAPP Oligomers

Immune system activation is suggested in DM1 due to several key findings related to immune responses to IAPP and beta cell-specific epitopes.

In humans, only a few beta cells-specific epitopes have been reported. By using algorithms to predict nonameric cellular peptides binding to the HLA-A*0201 allele, prepoIAPP 5–14 was identified as a potential epitope. Peripheral Blood Mononuclear Cells (PBMCs) were isolated from 18 DM1 patients and 9 control subjects (Table S1), and peptide recognition was evaluated using the Enzyme-Linked ImmunoSpot (ELISpot) assay. PBMCs from recent-onset patients responded to the peptide, with a higher number of cytotoxic T cells observed (Table S2) [8].

Another study applied an HLA epitope model and HLA class I peptide affinity algorithms to identify additional epitopes in 24 recent-onset DM1 patients and 11 non-diabetic controls (Table S1). Interferon-gamma (IFN-γ) ELISpot assays showed that the recent-onset patients secreted IFN-γ in response to peptides IAPP5, IAPP9, IGRP152 and IGRP215. The proportion of responsive varied for each peptide (IAPP5: 7 of 19, 37%; IAPP9: 8 of 19, 37%; IGRP152: 8 of 19, 42%; and IGRP215: 13 of 19, 68%) [9]. Finally, a study of 14 patients diagnosed with DM1 within the previous 2.5 years, using matrix-assisted algorithms and ELISpot assays, showed that three of the patients responded to ppIAPP (9–17) [21].

The role of CD8 T cells has also been explored. The response of CD8 T cells from recent-onset DM1 patients to IAPP was assessed. In vitro culture, ELISpot, and HLA tetramers revealed CD8 T cells with cytotoxic activity [22]. In animal studies, it was developed an I-Ag7 tetramer with high affinity for the KS20 peptide to study IAPP-reactive CD4 T cells in NOD mice. Significant numbers of KS20-positive CD4 T cells were detected in the pancreas of prediabetic and diabetic mice (Table S1) [11]. Similarly, it was explored the role of IAPP in autoimmune diabetes using the bitransgenic RIP-CD80xRIP-LCMV-GP (RIP-CD80GP) mouse model. DNA vaccination encoding IAPP induced diabetes in 25–33% of these mice, suggesting IAPP’s involvement in autoimmune diabetes development [14].

Finally, IAPP levels on plasma were analyzed. Plasma samples from 224 DM1 patients (Table S1) were analyzed at diagnosis, and IAPP levels exceeded 100 pmol/L in 25 patients (Table S2). Also, autoantibodies against IAPP were detected in 18% of patient samples using ELISA [2]. Additionally, pancreatic tissues from 10 DM1 patients (Table S1) were analyzed to investigate the reduction of IAPP-positive cells. 34% of islet cells were positive for IAPP. The ratio of IAPP-positive to insulin-positive cells was lower in diabetic islets than in controls [1].

### 3.5. Endoplasmic Reticulum Stress and Beta-Cell Dysfunction in DM1

The development of DM1 is influenced by genetic, cellular and environmental factors, one being the ER stress. ER stress expression in pancreatic cells leads to defects in protein processing, the generation of autoantigens and the enrichment of calcium-dependent channels in a feedback loop. Elevated ER stress levels also promotes the accumulation of misfolded proteins, such as IAPP, which further exacerbates ER stress [23]. Pancreatic beta cells are particularly vulnerable to excessive ER stress and dysregulated phosphorylation of elF2α, a key factor in the adaptive response to ER stress. Its dysregulation can accelerate beta-cell death [24]. Further insights into molecular mechanisms affecting beta cells in DM1 highlight immune-mediated cell death, including necroptosis and apoptosis associated with insulitis [25].

Comparative analyses of pancreatic tissues from individuals with DM1, DM2 and non-diabetic controls, as well as experiments with human IAPP (hIAPP) transgenic rats, reveal differences in the mechanisms of beta-cell apoptosis between DM1 and DM2. ER stress is a hallmark of DM2 but not DM1. Proposed triggers of beta-cell apoptosis in DM2 include prolonged exposure to elevated glucose or fatty acids. However, the absence of ER stress in the beta cells of humans with both long-standing and newly diagnosed DM1 challenges the notion that glucose or cytokine toxicity is the primary driver of beta-cell apoptosis in DM1 [18].

### 3.6. The Role of Beta Cell Apoptosis and Inflammatory Mechanisms in the Pathogenesis if DM1

Apoptosis is primarily triggered by autoreactive T cells and modulated by various mediators and effector molecules, including FasL, IL-1β, TNF-α, and IFN-γ [26]. The inflammatory properties of IAPP aggregates appear to contribute significantly to the chronic sterile inflammation observed in pancreatic islets. One study highlights that while 1L-1β synthesis occurs in most cell types, depletion of islet macrophages in vivo through clodronate virtually eliminates 1L-1β and TNF expression in hIAPP transgenic mice. Furthermore, hIAPP-induced 1L-1β release from bone marrow-derived macrophages (BMDMs) and dendritic cells (BMDCs) is dependent on phagocytosis of hIAPP aggregates, along with activation of NLRP3 and caspase 1. Deletion of either NLRP3 or caspase 1 abolishes hIAPP-induced IL-1β secretion in these cells [27].

### 3.7. Complementary Insights in Human Models: The Role of Gut Microbiome, Islet Transplantation and C-Peptide in IAPP Aggregation

#### 3.7.1. Role of Gut Microbiome and Bacterial Amyloids in DM1

Tetz et al. investigated 10 children with autoantibodies and 8 non-seroconverted controls, linking amyloid-producing E. coli in the gut with DM1-associated autoimmunity before seroconversion (Table S1). It is hypothesized that bacterial amyloids contribute to beta-cell destruction and the release of beta antigens, promoting IAPP aggregation and deposition in the pancreas [28].

#### 3.7.2. IAPP Amyloid Formation in Islet Transplantation

IAPP has a notable tendency to aggregate into amyloid deposits within pancreatic islets, a phenomenon particularly observed in DM2. These deposits exhibit cytotoxic properties and are a significant factor in beta-cell loss following islet transplants in DM1 patients [29]. Additional stressors, such as hyperglycemia and insulin resistance are known to exacerbate amyloid formation by increasing IAPP production. In transplanted islets, these stress conditions accelerate amyloid accumulation, impair proinsulin processing, and lead to a progressive decline in beta-cell function and mass [30].

Studies examining plasma levels of IAPP precursors in diabetic patients have shown that children with DM1 have lower plasma concentrations of mature IAPP and proIAPP-48 compared to healthy controls. However, the ratio of proIAPP-48 to total IAPP species is significantly elevated in DM1 patients [12] (Tables S1 and S2).

Further evidence highlights the presence of widespread amyloid deposits composed of IAPP in pancreatic islet grafts in diabetic patients. Congo red staining revealed amyloid accumulation in a substantial portion of recovered islets, with deposits spreading around capillaries and along the islet periphery. In one case, more than 40% of the examined islets contained amyloid deposits, illustrating the challenges associated with islet transplantation [3] (Tables S1 and S2). Complementary research indicates that while amyloid deposits are a common occurrence in pancreas transplants, they are not primarily caused by poor nutrition beneath the renal capsule [31].

#### 3.7.3. C-Peptide as a Modulator of Insulin and IAPP Aggregation

C-peptide plays a critical role in regulating protein aggregation associated with diabetes. It has been shown to exhibit a chaperone-like effect, reducing insulin fibrillogenesis and inhibiting IAPP aggregation. The absence of C-peptide in insulin-only treatments for DM1 patients likely contributes to the formation of protein deposits [32].

Advancements in understanding the molecular dynamics of C-peptide and insulin have provided compelling insights. Electrospray Ionization Mass Spectrometry (ESI-MS) has revealed direct interactions between the two molecules, while Hydrogen-Deuterium Exchange Mass Spectrometry (HDX-MS) showed that C-peptide reduces backbone accessibility in insulin fibrils. These findings highlight C-peptides significant role in mitigating IAPP aggregation [16].

### 3.8. Complementary Insights from Animal Studies on IAPP Aggregation, Toxicity and Therapeutic Approaches

#### 3.8.1. Effects of Heparin on IAPP Fibril Formation

Amyloid deposits in pancreatic islets present a significant obstacle to successful islet transplantation. Research has explored the role of heparin, a common anticoagulant used in clinical islet transplantation in influencing IAPP fibril formation. Observations using Transmission Electron Microscopy (TEM) confirmed that heparin promotes hIAPP fibril formation. Interestingly, as these fibrils mature, their cytotoxicity decreases. Moreover, heparin was unexpectedly found to reduce apoptosis in islet cells, suggesting a complex role in transplant outcomes [13].

#### 3.8.2. Comparison of Amyloidogenic Variants

The sequence of amylin is highly conserved among species, but small sequence variations strongly affect its amyloidogenic potential. For instance, bovine amylin, which differs from the human peptide at ten positions, forms non-toxic oligomers in beta cells. This observation highlights potential applications for designing non-amyloidogenic variants for therapeutic purposes. Insights from studies using the RIP-CD80GP mouse model, though not directly related to DM1, may provide valuable information for understanding the mechanisms underlying amyloidogenicity and its implications for future treatments [33].

#### 3.8.3. Assessment of Apoptosis in Animal Models

Fas-associated death receptor signaling has been identified as a pathway contributing to hIAPP-induced beta-cell apoptosis. Isolated mouse islets and insulinoma cell lines were analyzed to assess the expression of Fas, Fas ligand, and Fas-associated death domain (FADD) using techniques such as reverse transcription-polymerase chain reaction (RT-PCR), WB, and immunofluorescence (Table S1). Results demonstrated increased Fas and FADD expression during hIAPP-induced apoptosis. However, it remains unclear whether this pathway operates independently of endoplasmic reticulum stress [17].

In transgenic mice expressing hIAPP, a high-fat diet was shown to exacerbate diabetes-related symptoms. Treatment with the autophagy enhancer MSL-7 improved the metabolic profile by activating TFEB, promoting lysosomal biogenesis, and upregulating autophagy-related genes. This intervention enhanced beta-cell function, likely by facilitating the clearance of hIAPP oligomers and reducing beta-cell death [34].

### 3.9. Complementary Insights from Cellular Studies on IAPP-Induced Apoptosis and Therapeutic Strategies

#### 3.9.1. Role of Caspase-8 in Amyloid-Induced Beta-Cell Apoptosis

The role of caspase-8 in amyloid-induced beta-cell apoptosis has been investigated using human and mouse islets expressing hIAPP with beta-cell-specific caspase-8 deletion. Evidence suggests that amyloid-induced overexpression of Fas in beta cells promotes caspase-8 activation, a key mediator of apoptosis. Furthermore, inhibiting oligomers with Congo red significantly reduced cell death, indicating potential therapeutic value [35].

#### 3.9.2. Protective Effects of p35 on Beta-Cell Viability

Inflammatory cytokines were used to induce diabetes in beta cells, simulating the autoimmune environment of the disease. The protein p35 demonstrated a protective effect by inhibiting apoptosis caused by both cytokines and hIAPP. Results from MTT assays indicated increased cell viability, while TUNEL assays and caspase activity measurements confirmed reduced apoptosis. Additionally, p35 enhanced resistance to hIAPP-induced beta-cell death, highlighting its potential as a therapeutic agent [36].

#### 3.9.3. Exploring Pramlintide as an Adjunct Therapy in DM1

Despite advancements in insulin therapy, complementary treatments are needed to address persistent challenges in glucose regulation. IAPP plays a synergistic role with insulin in glucose control, and pramlintide, an amylin analog, has been explored as an adjunct therapy for DM1. Studies indicate that pramlintide can counteract insulin-induced hypoglycemia and regulate hyperglycemic peaks by mimicking amylin’s physiological effects [37,38].

However, pramlintide use is not without challenges. Adverse gastrointestinal effects, such as nausea, vomiting, and diarrhea, were dose-limiting in clinical trials. Severe hypoglycemia also led to treatment discontinuation in some cases. Nonetheless, evidence supports its compatibility for co-administration with insulin in the same syringe without adverse effects. These findings highlight pramlintide’s potential to improve glucose control while mitigating risks associated with insulin therapy [37,38].

**Figure 7:**
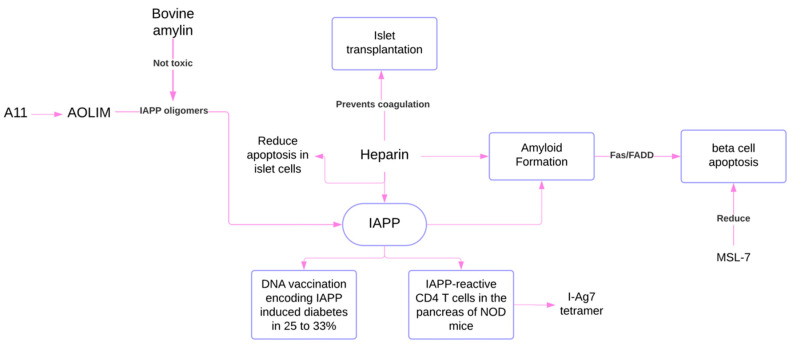
Animal Models of IAPP Toxicity. This diagram illustrates several experimental strategies aimed at mitigating the detrimental effects of IAPP in animal models of diabetes. These strategies include using non-toxic bovine amylin as a substitute for human IAPP and employing heparin to prevent coagulation and reduce apoptosis in islet cells. Additionally, the diagram highlights the potential role of the immune system in IAPP-related disease, as evidenced by the induction of diabetes in some animal models through DNA vaccination encoding IAPP and the presence of IAPP-reactive CD4 T cells in the pancreas of certain mice strains. These findings, while derived from animal studies, offer valuable insights into potential therapeutic approaches for addressing the complications associated with IAPP-related beta cell dysfunction and death in diabetes.

**Figure 8:**
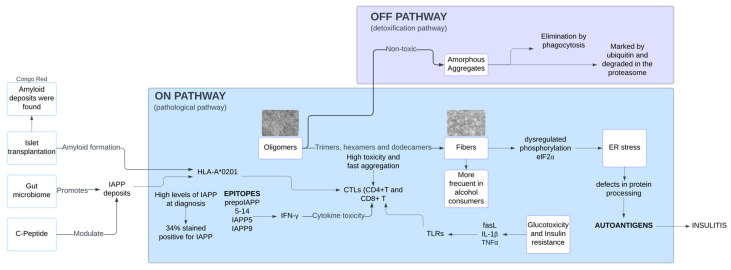
Pathogenesis of DM1. This diagram depicts the intricate pathways involved in DM1. It highlights the central role of islet transplantation in the disease process and the presence of amyloid deposits. The diagram illustrates how the gut microbiome, insulin resistance, and the immune system all contribute to the pathogenesis of the disease. Key features include the formation of amyloid from IAPP (islet amyloid polypeptide), the impact of insulin resistance on glucotoxicity and cytokine production, and the involvement of the immune system in autoimmune responses against islet cells.

The following figures were created to connect the results obtained in the study. They visually and clearly represent the relationship between the data, facilitating the interpretation of the findings and highlighting key patterns (Figure 7 and Figure 8).

### 3.10. Differences and Similarities Between DM1 and DM2

IAPP aggregation follows a complex dynamic, starting with the formation of hexamers, then oligomers and finally amyloid fibers, however, it is important to mention that these fibers are more associated with DM2, but also play an important role in DM1. Although the formation of these structures is characteristic of both conditions, the context and pathological implication vary between them.

Both conditions are associated with pancreatic beta-cell dysfunction and endoplasmic reticulum stress, which contributes to cell damage and hyperglycemia. However, DM1 is an autoimmune disease in which the immune system attacks beta cells, progressively destroying them. This attack is not only a result of metabolic dysfunction but also of an exacerbating immune response. In this context, the accumulation of IAPP is not only a result of cellular damage but may also act as a trigger or amplifier of the autoimmune response. The accumulation of IAPP and its subsequent aggregation can create a cycle in which IAPP itself promotes an inflammatory environment that perpetuates damage and destruction of beta cells.

In contrast, DM2 is characterized by insulin resistance and progressive beta dysfunction. Insulin resistance occurs when the body’s cells do not respond properly to insulin, leading to elevated blood sugar levels. The formation of IAPP fibers is more related to chronic inflammation, oxidative stress and proteasome dysfunction. These mechanisms are linked to protein homeostasis and cellular function. Chronic inflammation and metabolic stress exacerbate beta cell dysfunction and contribute to insulin resistance. This can lead to a feedback loop where elevated blood glucose further damage the pancreas, worsening the disease [25].

**Figure 9:**
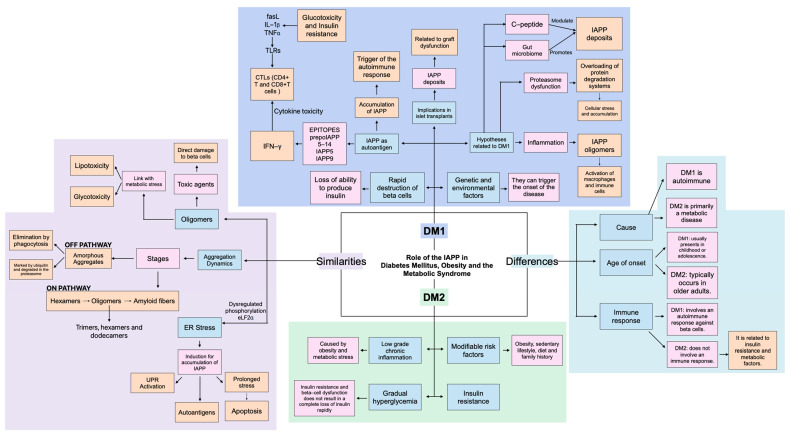
Role of IAPP in Type 1 and Type 2 Diabetes. This figure explores the role of IAPP in the pathogenesis of DM1 and DM2. In DM1, IAPP acts as a trigger for the autoimmune response, with its accumulation and recognition by T lymphocytes contributing to beta cell destruction. The figure outlines the aggregation dynamics of IAPP, progressing from hexamers to toxic oligomers and amyloid fibers, which induce ER stress, activate the unfolded protein response (UPR), and lead to beta cell apoptosis. It also emphasizes the involvement of IAPP in inflammation, proteasome dysfunction, and its implications in islet transplants. Comparatively, DM2 is characterized as a metabolic disease driven by obesity and insulin resistance, with low-grade chronic inflammation and gradual beta cell dysfunction. While DM1 is primarily autoimmune and presents in childhood or adolescence, DM2 typically occurs in older adults and lacks an immune-mediated mechanism. Shared mechanisms include metabolic stress and IAPP aggregation, but their outcomes differ significantly between the diseases.

Although DM1 and DM2 share certain pathological characteristics, the mechanisms in which IAPP aggregation occurs differ significantly between the two. In DM1, amyloid fiber formation may be less prominent that in DM2, yet it still plays a meaningful role in disease progression. While both disorders exhibit overlapping pathways, they retain distinctive features: DM2 is primarily driven by insulin resistance, whereas DM1 stems from autoimmune destruction of beta cells (Figure 9).

## 4. Discussion

The findings of this systematic review and epistemic meta-analysis (Figure 3, Figure 4, Figure 5, Figure 6, Figure 7, Figure 8 and Figure 9). highlight the multifaceted role of IAPP aggregation in the pathogenesis of DM1, providing compelling evidence for its involvement in beta-cell dysfunction and immune-mediated destruction. The evidence supports the hypothesis that IAPP aggregation acts not only as a pathological feature but also as a potential driver of autoimmune responses through interconnected mechanisms, including toxic oligomer formation, immune activation via epitope presentation and exacerbation of beta-cell stress, apoptosis, proteasome dysfunction and the promotion of chronic inflammation.

### 4.1. IAPP Aggregation and Beta-Cell Dysfunction

**Figure 10:**
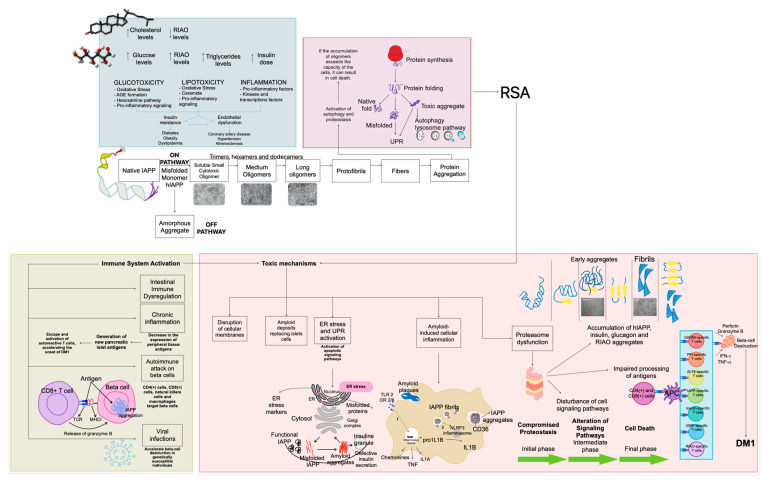
Mechanisms of IAPP aggregation and beta-cell dysfunction in DM1. Overview of the mechanisms involved in IAPP aggregation and its role in DM1 pathogenesis. The diagram integrates processes such as glucotoxicity, lipotoxicity, inflammation, membrane disruption, proteasome dysfunction, ER stress and immune system activation. The stages of IAPP aggregation are depicted (aggregation-oligomerization-fibrillization process). Together, this mechanisms conclude in progressive beta-cell dysfunction and death.

The discovery of toxic IAPP oligomers has been essential in understanding the subsequent mechanisms driving DM1 pathogenesis (Figure 1, Figure 2, Figure 3, Figure 4, Figure 5, Figure 6, Figure 7, Figure 8 and Figure 9). These oligomers and amyloid deposits mark the initial step in a cascade of damaging effects. Soluble oligomers, such as trimers and hexamers, exhibit higher cytotoxicity compared to larger aggregates, directly disrupting cellular membranes, impairing beta-cell function, and accelerating apoptosis [4,5]. For instance, soluble oligomers detected in DM1 serum samples displayed diverse aggregation states, and TEM confirmed their rapid progression into fibrillar amyloids, consistent with the amyloid cascade hypothesis. Importantly, these oligomers represent the “on-pathway” phase of the aggregation process; during this phase, small IAPP monomers undergo nucleation and progressively form intermediate oligomers, which serve as precursors to fibril growth (Figure 1, Figure 2, Figure 3, Figure 4, Figure 5, Figure 6, Figure 7, Figure 8, Figure 9 and Figure 10). These intermediate states are particularly toxic because of their ability to interact with and destabilize cellular membranes, causing ion leakage, mitochondrial dysfunction and activation of stress pathways in beta cells. In contrast, the “off-pathway” phase involves the aggregation of oligomers into larger, insoluble fibrils and amyloid plaques. Although these fibrillar structures are less cytotoxic compared to soluble oligomers, they contribute to the pathogenesis by inducing chronic inflammation and impairing islet architecture (Figure 8 and Figure 10).

This transition from soluble to insoluble forms not only exacerbates cellular stress but also alters the inflammatory and immune responses. This cytotoxicity is further exacerbated by glucotoxicity and lipotoxicity, suggesting that metabolic stress accelerates IAPP aggregation [5]. These events together initiate the release of reactive oxygen species (ROS), destabilizing cellular homeostasis and sensitizing beta cells to apoptosis-inducing signals.

Additionally, it is suggested that environmental factors may contribute to amyloid formation independently of immune response mechanisms. Bruggeman et al. demonstrated that substance use (alcohol, tobacco, marijuana, and illicit substances) impacted directly islet amyloid deposition [7], with alcohol users exhibiting a higher frequency of amyloid formation, supporting this hypothesis.

### 4.2. Immune Activation and Epitope Presentation

Several studies have identified IAPP-derived epitopes that activate cytotoxic T lymphocytes (CTLs), linking amyloid formation with the autoimmune component of DM1. For instance, preproIAPP (5–14) and IAPP5 and IAPP9 have been shown to get strong immune responses in HLA-A*0201-positive subjects [8,9] (Figure 8). The presence of reactive CD8+ T cells targeting these epitopes suggests that IAPP aggregation may contribute to the generation of autoantigens. This is supported by Peakman, who demonstrated that beta-cell specific epitopes (including those derived from IAPP), stimulate cytotoxic CD8+ T cell responses in DM1 patients [22]. This activation likely involves the interaction of T cell receptors (TCRs) on CD8+ T cells with the IAPP-MHC class I complex (Figure 10).

This immune activation may not only target beta cells but also perpetuate inflammation within the islets, forming a feedback loop between immune activation and IAPP aggregation.

### 4.3. Endoplasmic Reticulum Stress

ER stress is a critical factor in beta-cell dysfunction and appears to be closely linked to IAPP aggregation. Accumulation of IAPP aggregates in beta cells further exacerbates ER stress, triggering the unfolded protein response (UPR) and ER-associated protein degradation (ERAD). Studies have shown that dysregulated UPR contributes to beta-cell apoptosis, creating a feedback loop that further accelerates IAPP aggregation [24,25].

The UPR initially acts as a protective mechanism by upregulating chaperone proteins, such as BiP, and phosphorylating eIF2α [23] to reduce global translation. However, when these adaptive responses fail to restore proteostasis, the terminal UPR is activated, leading to increased expression of pro-apoptotic factors like CHOP, ultimately driving beta-cell apoptosis. Notably, studies in DM1 have reported elevated levels of ATF3, CHOP, and BiP in insulitis-positive islets [24], although variability in CHOP expression across different patients suggests the influence of genetic and environmental factors.

The apoptotic pathways involved in beta-cell death are both intrinsic and extrinsic. Intrinsic pathways are mediated by Bcl-2 family proteins, while extrinsic pathways involve death receptors like Fas, which are activated by ligands such as TNF-α [25]. Amyloid aggregates of IAPP can independently trigger beta-cell apoptosis via Fas signaling, bypassing the classical autoimmune mechanisms. Additionally, apoptosis and subsequent necrosis of beta cells release damage-associated molecular patterns (DAMPs), amplifying inflammation and potentially initiating or exacerbating autoimmune responses against beta cells.

A feedback loop may exist wherein ER stress increases IAPP levels, promoting further amyloid aggregation and worsening stress (Figure 10). This cycle perpetuates beta-cell dysfunction, apoptosis, and inflammation. The genetic predisposition to DM1, particularly through MHC loci like HLA class I and II alleles, further compounds the autoimmune response by presenting autoantigens to T cells, driving the destruction of beta cells [23]. Together, these mechanisms underscore the pivotal role of ER stress, IAPP aggregation, and apoptotic pathways in the pathogenesis of DM1, linking cellular dysfunction to the onset and progression of the disease.

### 4.4. Inflammation and Cytokine Release

Aggregated IAPP fibrils have been shown to activate the NLRP3 inflammasome in macrophages, leading to the secretion of pro-inflammatory cytokines such as interleukin-1 beta (1L-1β) [27] (Figure 10). The process of IL-1β release from BMDMs and BMDCs depends on the phagocytosis of hIAPP aggregates and activation of the NLRP3 inflammasome. Upon phagocytosis, these aggregates are recognized by pattern recognition receptors, leading to inflammasome activation and IL-1β release. Different aggregate species of hIAPP possess varying inflammatory potentials. Pre-fibrillar hIAPP species are particularly pro-inflammatory and toxic to INS1 cells, while fibrillar hIAPP can activate the inflammasome in LPS-primed macrophages. However, the specific species that induces the strongest inflammatory response remains undefined and an even bigger mystery is if IAPP-induced inflammation play a role in DM1, however it seems plausible that hIAPP aggregates could be a trigger or accelerator of autoimmunity in DM1, as was reported amyloid from pancreas biopsies. This raises the hypothesis that IAPP aggregation not only contributes to beta-cell dysfunction through direct toxicity but could also stimulate the immune system by enhancing the presentation of autoantigens or activating autoreactive immune cells. The chronic inflammation induced by IAPP aggregation may promote the escape and activation of autoreactive T cells, potentially accelerating the onset and progression of DM1.

### 4.5. Proteasome Dysfunction

Proteasome dysfunction is suggested to play a role in DM1. Although proteasome dysfunction was not reported in any of the articles obtained from the databases, we considered it important to include this aspect due to its potential relevance in the pathophysiology of DM1. The proteasome is a critical component of cellular protein homeostasis, and its dysfunction could amplify the effects of IAPP misfolding, linking it to mechanisms that may underlie beta-cell failure and autoimmunity in DM1.

When proteasomal activity is impaired, the accumulation of misfolded IAPP overwhelms the cellular quality control systems, exacerbating toxic oligomer formation and promoting aggregation-prone intermediates. This dysfunction shifts the balance of IAPP aggregation toward the “on-pathway” formation of cytotoxic oligomers and fibrils, while also increasing the probability of “off-pathway” aggregates that contribute to cellular stress (Figure 8).

One major impact of proteasome dysfunction is the disruption of antigen presentation. The impaired degradation of misfolded IAPP may enhance its presentation as aberrant epitopes, triggering autoimmune responses. In addition, impaired proteasome activity disturbs cell signaling networks that control cell growth, apoptosis, immunity and other factors [39] (Figure 10).

The proteasomal backlog increases the burden on the cellular machinery, such as the ER, intensifying ER stress and further propagating the feedback loop of beta-cell dysfunction and apoptosis. This dysfunction also contributes to inflammation by failing to clear proteins such as aggregated IAPP fibrils, which have been shown to activate the NLRP3 inflammasome in macrophages.

### 4.6. Connecting the Mechanisms

The interplay between these mechanisms underscores the complexity of the role of IAPP in DM1. IAPP aggregation directly damages beta cells through membrane disruption and ROS generation while simultaneously creating conditions that facilitate immune recognition of beta-cell antigens. Proteasome dysfunction further amplifies these effects by impairing the degradation of misfolded IAPP and promoting the accumulation of toxic intermediates. This immune activation is further amplified by ER stress, which not only enhances aggregation but also generates additional autoantigens, intensifying the autoimmune response. The formation of oligomers, protofibrils and fibers contribute to cellular toxicity. The toxic mechanisms disrupt proteostasis, activating autophagy. This creates a cycle where metabolic stressors, such as glucotoxicity, lipotoxicity and chronic inflammation exacerbate the burden on beta cells (Figure 1, Figure 4, Figure 8, Figure 9 and Figure 10). The resultant pro-inflammatory environment driven by cytokine release establishes a chronic cycle of damage, immune activation, and cellular stress. These interconnected processes highlight how metabolic dysfunction and immune mechanisms converge to accelerate beta-cell failure in DM1 (Figure 10).

### 4.7. Exploring Amyloid Aggregation and Contributing Factors in Islet Transplants and Beta-Cell Destruction

The role of amyloid aggregation in islet transplant has been extensively studied. Several studies have demonstrated the presence of IAPP deposits in islet transplants. Westermark et al. reported that amyloid deposits were identified in human islet grafts transplanted into nude mice, with Congo Red staining confirming their amyloid nature. TEM later revealed that the deposits were associated with IAPP [29]. This align with observations in transgenic mice expressing hIAPP, where amyloid deposition was associated with increased beta-cell apoptosis [31].

**Figure 11:**
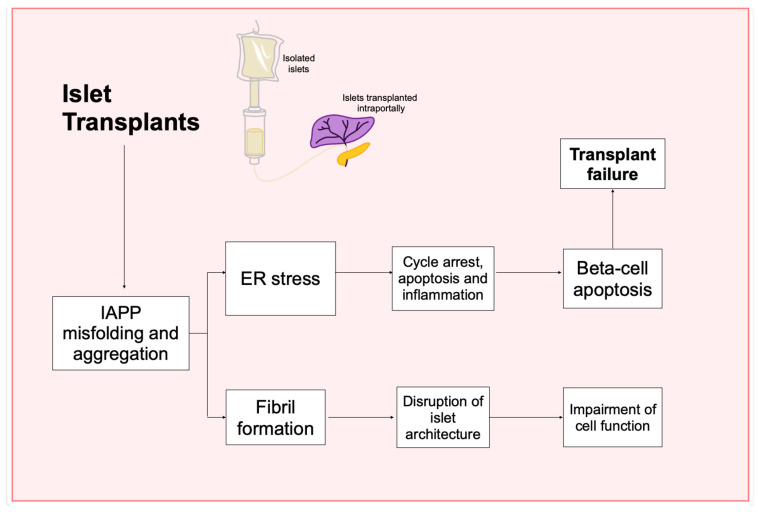
Amyloid aggregation in islet transplant. This diagram depicts the relation between IAPP aggregation and beta-cell function in islet transplants. It starts with islet transplants, which initiate a sequence of harmful events when IAPP misfolds and aggregates. The misfolding results in the formation of amyloid fibrils, which disrupt the islet architecture, then impairs the function of the cells within these islets. The misfolding of IAPP also causes ER stress, leading to cellular cycle arrest, inflammation and beta-cell apoptosis; As this progresses, it results in the failure of the islet transplant.

The rapid formation of amyloid deposits further underscores their potential to impaired beta-cell function. Additionally, it was revealed that IAPP aggregates in transplanted islets can trigger sterile inflammation [27] (Figure 11), however, the temporal aspect of amyloid formation adds complexity to its role. Westermark et al. observed variability in amyloid deposits among islet-bearing recipients, suggesting that amyloid formation is a time-dependent process [3].

Beyond IAPP, other factors such as bacterial amyloids and C-peptide also influence amyloid aggregation. Tetz et al. proposed that bacterial amyloids produced by E. coli may stimulate IAPP aggregation and deposition in the pancreas. Although the exact mechanisms remain unclear, the potential role of bacterial amyloids in beta-cell destruction needs more investigation [28].

**Figure 12:**
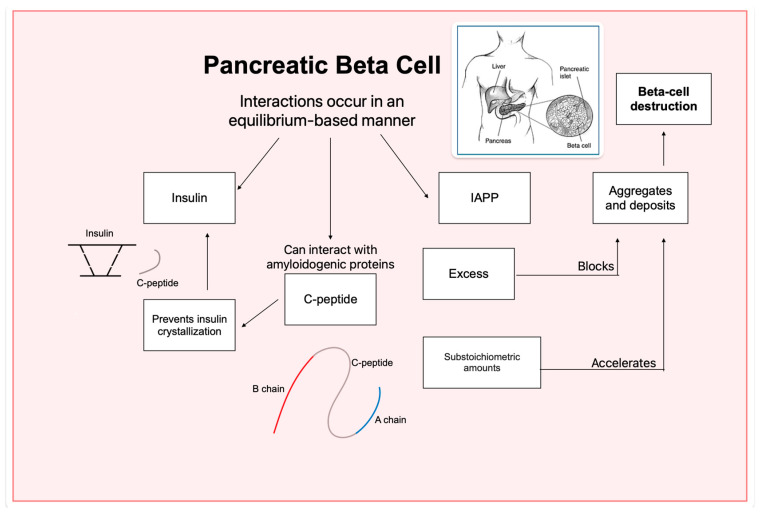
Modulation of Insulin and IAPP by C-peptide interactions. This diagram illustrates the interactions between C-peptide, insulin and IAPP in the context of fibril formation. Insulin coexists with IAPP and the C-peptide is released together with insulin. The C-peptide prevents the formation of insulin crystals, however, under certain conditions, it can interact with amyloid proteins and accelerate the formation of toxic aggregates. There is a delicate balance between insulin, IAPP and C-peptide, and any alteration, such as excess IAPP can trigger the development of diabetes.

C-peptide has been identified as a modulator of IAPP aggregation and fibrillation. Substoichiometric amounts of C-peptide promote IAPP self-association, while excess of C-peptide prevents aggregation by saturating binding sites on IAPP molecules (Figure 12). This interactions emphasize the balance required to prevent aggregation while maintaining normal protein function within beta-cell granules [16,32]. 

### 4.8. Target Biomarkers in DM1

RIAO and IAPP hexamers can be considered target biomarkers in DM1 due to their involvement in the pathological processes characteristic of the disease. The presence of RIAO is associated with toxicity to beta cells, and it’s detection could reflect the pathological state of beta cells; RIAO also may stimulate the immune system, so their measurement could provide insights into autoimmune activity, and it is also related to ER stress, indicating the level of cellular stress.

On the other hand, IAPP hexamers could reflect the state of protein in the context of diabetes. Their structures may precede the formation of more toxic aggregates, so their level in plasma could indicate the state of the protein. Also, hexamers can be recognized by the immune system, making them biomarkers to asses autoimmunity.

### 4.9. New Insights into the Role of IAPP Oligomers in DM1

RIAO levels correlate with beta-cell failure and suggested that these oligomers may be early precursors of amyloid fibers, and most importantly, RIAO levels were influenced by glucose and cholesterol levels, showing a novel connection between IAPP oligomers, glucotoxicity and lipotoxicity in DM1. It was also suggested a novel mechanism of the disease progression; the prion-like transmission of IAPP oligomers could play a role in the progressive nature of DM1 (Figure 1, Figure 8, Figure 9 and Figure 10). The spread misfolded proteins may act as a trigger for increased immune responses, chronic inflammation, ending in beta-cell damage.

Research has revealed that amyloid deposits in human islet grafts play a significant role in beta-cell dysfunction, with the rapid formation of aggregated triggering sterile inflammation that jeopardizes transplant success. The contribution of IAPP to sterile inflammation is further highlighted by its ability to activate immune cells, leading to the release of pro-inflammatory cytokines. This inflammatory response is initiated through the phagocytosis of IAPP aggregates and the subsequent activation of the NLRP3 inflammasome.

Finally, it was presented a relation between bacterial amyloids, specifically from E. coli, with beta-cell destruction through IAPP aggregation, suggesting a possible microbial influence in DM1 pathogenesis.

### 4.10. Impact and Future Direction of Research on IAPP Oligomers in DM1

The connection between IAPP oligomers and beta-cell toxicity presents new opportunities to develop therapies that prevent the formation of toxic oligomers. IAPP oligomers play a crucial role in the pathogenesis of diabetes mellitus, particularly in the destruction of pancreatic beta cells and the dysfunction of insulin production. The formation of these oligomers, along with their ability to induce apoptosis and alter cellular function, highlights the importance of IAPP aggregation and oligomerization processes in disease progression. The link between glucotoxicity, lipotoxicity, and cholesterol with IAPP further provides insights into how metabolic alterations can exacerbate diabetes. Additionally, hIAPP has emerged as a potential biomarker for the early diagnosis of diabetes, allowing the identification of the disease before the onset of clinical symptoms (Figure 1, Figure 4, Figure 8, Figure 9 and Figure 10).

Research on prion-like transmission and amyloid accumulation in transplants may change the approach to islet transplant-based treatments and enhance our understanding of disease progression in transplant recipients. However, more research is essential to translate these findings into effective clinical treatments.

Although the role of IAPP oligomers in cellular damage in DM1 is not fully understood, further research is needed to elucidate the mechanisms of oligomer formation and propagation. Modulating the formation of IAPP oligomers and fibrils could lead to strategies that preserve beta-cell function and slow disease progression. However, research remains crucial to effectively address the harmful effects of these protein aggregates in diabetes mellitus.

### 4.11. Limitations of the Study

There are conflicting findings regarding the presence of amyloid deposits, suggesting that the underlying mechanisms may differ among patient subpopulations or at various stages of the disease. Additionally, many studies have been conducted on small samples or animal models, which may not fully capture the progression of DM1 in humans. Therefore, clinical trials in human subjects are essential to confirm the role of IAPP oligomers and asses potential therapeutic interventions in a clinical setting.

## 5. Conclusions

IAPP oligomers have been identified in patients with DM1 through various techniques, including Western Blot (WB), Transmission Electron Microscopy (TEM), Thioflavin (ThT) fluorescence, Congo red staining, ELISA, and immunocytochemical staining. These toxic oligomers have been detected in pancreatic islets of both human and animal models, where they appear to play a significant role in beta cell damage and contribute to the progression of DM1. The identification of IAPP oligomers as toxic entities in DM1 advances our understanding of its pathogenesis and paves the way for innovative diagnostic and therapeutic strategies.

## 6. Image Credit

The images used in Figure 8 and Figure 10 were obtained from the following articles: Diabetes Drug Discovery: hIAPP (1–37) Polymorphic Amyloid Structures as Novel Therapeutic Targets, published in MDPI in 2018; Unpacking the aggregation-oligomerization-fibrillization process of naturally-occurring hIAPP amyloid oligomers isolated directly from sera of children with obesity or diabetes mellitus, published in SCIENTIFIC REPORTS in 2019; and Protein-conformational diseases in childhood: Naturally-occurring hIAPP amyloid-oligomers and early β-cell damage in obesity and diabetes, published in PLOS ONE in 2020. These images were used with the permission of Myriam M. Altamirano-Bustamante.

## Data Availability

This study did not created or analyzed new data.

## Supplementary Materials

The following supporting information can be downloaded at: https://www.mdpi.com/article/10.3390/ijms26020767/s1.

## References

[1] Tomita T. (2011). Islet amyloid polypeptide in pancreatic islets from type 1 diabetic subjects. Islets.

[2] Paulsson J.F., Ludvigsson J., Carlsson A., Casas R., Forsander G., Ivarsson S.A., Kockum I., Lernmark Å., Marcus C., Lindblad B. (2014). High plasma levels of islet amyloid polypeptide in young with new-onset of type 1 diabetes mellitus. PLoS ONE.

[3] Westermark G.T., Davalli A.M., Secchi A., Folli F., Kin T., Toso C., Shapiro A.M.J., Korsgren O., Tufveson G., Andersson A. (2012). Further evidence for amyloid deposition in clinical pancreatic islet grafts. Transplantation.

[4] Altamirano-Bustamante M.M., Altamirano-Bustamante N.F., Larralde-Laborde M., Lara-Martínez R., Leyva-García E., Garrido-Magaña E., Rojas G., Jiménez-García L.F., Revilla-Monsalve C., Altamirano P. (2019). Unpacking the aggregation-oligomerization-fibrillization process of naturally-occurring hIAPP amyloid oligomers isolated directly from sera of children with obesity or diabetes mellitus. Sci. Rep..

[5] Altamirano-Bustamante N.F., Garrido-Magaña E., Morán E., Calderón A., Pasten-Hidalgo K., Castillo-Rodríguez R.A., Rojas G., Lara-Martínez R., Leyva-García E., Larralde-Laborde M. (2020). Protein-conformational diseases in childhood: Naturally-occurring hIAPP amyloid-oligomers and early β-cell damage in obesity and diabetes. PLoS ONE.

[6] Beery M.L., Jacobsen L.M., Atkinson M.A., Butler A.E., Campbell-Thompson M. (2019). Islet amyloidosis in a child with type 1 diabetes. Islets.

[7] Bruggeman B.S., Campbell-Thompson M., Filipp S.L., Gurka M.J., Atkinson M.A., Schatz D.A., Jacobsen L.M. (2021). Substance Use Affects Type 1 Diabetes Pancreas Pathology: Implications for Future Studies. Front. Endocrinol..

[8] Panagiotopoulos C., Qin H., Tan R., Verchere C.B. (2003). Identification of a beta-cell-specific HLA class I restricted epitope in type 1 diabetes. Diabetes.

[9] Ouyang Q., Standifer N.E., Qin H., Gottlieb P., Verchere C.B., Nepom G.T., Tan R., Panagiotopoulos C. (2006). Recognition of HLA class I-restricted beta-cell epitopes in type 1 diabetes. Diabetes.

[10] Zhang S., Liu H., Chuang C.L., Li X., Au M., Zhang L., Phillips A.R.J., Scott D.W., Cooper G.J.S. (2014). The pathogenic mechanism of diabetes varies with the degree of overexpression and oligomerization of human amylin in the pancreatic islet β cells. FASEB J..

[11] Baker R.L., Delong T., Barbour G., Bradley B., Nakayama M., Haskins K. (2013). Cutting edge: CD4 T cells reactive to an islet amyloid polypeptide peptide accumulate in the pancreas and contribute to disease pathogenesis in nonobese diabetic mice. J. Immunol..

[12] Courtade J.A., Klimek-Abercrombie A.M., Chen Y.-C., Patel N., Lu P.Y.T., Speake C., Orban P.C., Najafian B., Meneilly G., Greenbaum C.J. (2017). Measurement of Pro-Islet Amyloid Polypeptide (1–48) in Diabetes and Islet Transplants. J. Clin. Endocrinol. Metab..

[13] Potter K.J., Werner I., Denroche H.C., Montane J., Plesner A., Chen Y., Lei D., Soukhatcheva G., Warnock G.L., Oberholzer J. (2015). Amyloid formation in human islets is enhanced by heparin and inhibited by heparinase. Am. J. Transplant..

[14] Fuchs Y.F., Adler K., Lindner A., Karasinsky A., Wilhelm C., Weigelt M., Balke H., Förtsch K., Mortler-Hildebrandt L.F., Harlan D.M. (2014). IGRP and insulin vaccination induce CD8+ T cell-mediated autoimmune diabetes in the RIP-CD80GP mouse. Clin. Exp. Immunol..

[15] Lv Y.-Q., Yuan L., Sun Y., Dou H.-W., Su J.-H., Hou Z.-P., Li J.-Y., Li W. (2022). Long-term hyperglycemia aggravates α-synuclein aggregation and dopaminergic neuronal loss in a Parkinson’s disease mouse model. Transl. Neurodegener..

[16] Landreh M., Stukenborg J.-B., Willander H., Söder O., Johansson J., Jörnvall H. (2012). Proinsulin C-peptide interferes with insulin fibril formation. Biochem. Biophys. Res. Commun..

[17] Zhang S., Liu H., Yu H., Cooper G.J.S. (2008). Fas-associated death receptor signaling evoked by human amylin in islet beta-cells. Diabetes.

[18] Huang C.-j., Lin C.-y., Haataja L., Gurlo T., Butler A.E., Rizza R.A., Butler P.C. (2007). High expression rates of human islet amyloid polypeptide induce endoplasmic reticulum stress mediated beta-cell apoptosis, a characteristic of humans with type 2 but not type 1 diabetes. Diabetes.

[19] Aida K., Fukui T., Jimbo E.B., Yagihashi S., Shimada A., Oikawa Y., Mori Y., Fujii T., Nishida Y., Koyama R. (2018). Distinct Inflammatory Changes of the Pancreas of Slowly Progressive Insulin-dependent (Type 1) Diabetes. Pancreas.

[20] Leyva-García E., Lara-Martínez R., Morán-Zanabria L., Revilla-Monsalve C., Jiménez-García L.F., Oviedo N., Murata C., Garrido-Magaña E., Altamirano-Bustamante N.F., Altamirano-Bustamante M.M. (2017). Novel insight into streptozotocin-induced diabetic rats from the protein misfolding perspective. Sci. Rep..

[21] Standifer N.E., Ouyang Q., Panagiotopoulos C., Verchere C.B., Tan R., Greenbaum C.J., Pihoker C., Nepom G.T. (2006). Identification of Novel HLA-A*0201-restricted epitopes in recent-onset type 1 diabetic subjects and antibody-positive relatives. Diabetes.

[22] Peakman M. (2008). CD8 and cytotoxic T cells in type 1 diabetes. Defining Optimal Immunotherapies for Type 1 Diabetes: Novartis Foundation Symposium 292.

[23] Makam A.A., Biswas A., Kothegala L., Gandasi N.R. (2022). Setting the Stage for Insulin Granule Dysfunction during Type-1-Diabetes: Is ER Stress the Culprit?. Biomedicines.

[24] Cnop M., Toivonen S., Igoillo-Esteve M., Salpea P. (2017). Endoplasmic reticulum stress and eIF2α phosphorylation: The Achilles heel of pancreatic β cells. Mol. Metab..

[25] Rojas J., Bermudez V., Palmar J., Martínez M.S., Olivar L.C., Nava M., Tomey D., Rojas M., Salazar J., Garicano C. (2018). Pancreatic Beta Cell Death: Novel Potential Mechanisms in Diabetes Therapy. J. Diabetes Res..

[26] Quan W., Jo E., Lee M. (2013). Role of pancreatic *β*-cell death and inflammation in diabetes. Diabetes, Obes. Metab..

[27] Denroche H.C., Verchere C.B. (2018). IAPP and type 1 diabetes: Implications for immunity, metabolism and islet transplants. J. Mol. Endocrinol..

[28] Tetz G., Brown S.M., Hao Y., Tetz V. (2019). Type 1 Diabetes: An Association Between Autoimmunity, the Dynamics of Gut Amyloid-producing *E. coli* and Their Phages. Sci. Rep..

[29] Westermark P., Andersson A., Westermark G.T. (2011). Islet amyloid polypeptide, islet amyloid, and diabetes mellitus. Physiol. Rev..

[30] Montane J., Klimek-Abercrombie A., Potter K.J., Westwell-Roper C., Verchere C.B. (2012). Metabolic stress, IAPP and islet amyloid. Diabetes Obes. Metab..

[31] Westermark P., Andersson A., Westermark G.T. (2005). Is aggregated IAPP a cause of beta-cell failure in transplanted human pancreatic islets?. Curr. Diabetes Rep..

[32] Landreh M., Johansson J., Jörnvall H. (2013). C-peptide: A molecule balancing insulin states in secretion and diabetes-associated depository conditions. Horm. Metab. Res..

[33] Akter R., Bower R.L., Abedini A., Schmidt A.M., Hay D.L., Raleigh D.P. (2018). Amyloidogenicity, Cytotoxicity, and Receptor Activity of Bovine Amylin: Implications for Xenobiotic Transplantation and the Design of Nontoxic Amylin Variants. ACS Chem. Biol..

[34] Kim J., Park K., Kim M.J., Lim H., Kim K.H., Lee E.-S., Kim H., Kim S.J., Hur K.Y., Kim J.H. (2021). An autophagy enhancer ameliorates diabetes of human IAPP-transgenic mice through clearance of amyloidogenic oligomer. Nat. Commun..

[35] Park Y.J., Woo M., Kieffer T.J., Hakem R., Safikhan N., Yang F., Ao Z., Warnock G.L., Marzban L. (2014). The role of caspase-8 in amyloid-induced beta cell death in human and mouse islets. Diabetologia.

[36] Hollander K., Bar-Chen M., Efrat S. (2005). Baculovirus p35 increases pancreatic β-cell resistance to apoptosis. Biochem. Biophys. Res. Commun..

[37] George P., McCrimmon R.J. (2013). Potential role of non-insulin adjunct therapy in Type 1 diabetes. Diabet. Med..

[38] (2003). Pramlintide: (AC 137, AC 0137, Symlin, Tripro-Amylin). BioDrugs.

[39] Dikic I. (2017). Proteasomal and Autophagic Degradation Systems. Annu. Rev. Biochem..

