# Supplementary material for: Decoding the Contribution of IAPP Amyloid Aggregation to Beta Cell Dysfunction: A Systematic Review and Epistemic Meta-Analysis of Type 1 Diabetes
Source: Int J Mol Sci. 2025 Jan 17;26(2):767. doi: 10.3390/ijms26020767 (PMC11766435; doi:10.3390/ijms26020767)
Supplement: Supplementary file 1 [file ijms-26-00767-s001.zip › ijms-3384084-supplementary.pdf]

Supplementary material

Title of the article: Decoding the Contribution of IAPP Amyloid Aggregation to Beta Cell Dysfunction: A Systematic Review and Meta-Analysis of Type 1 Diabetes

Authors: Valeria Moya-Gudiño, Nelly F. Altamirano-Bustamante, Cristina Revilla-Monsalve and Myriam M. Altamirano-Bustamante

Affiliations: Unidad de Investigación en Enfermedades Metabólicas, Centro Médico Nacional Siglo XXI, IMSS, Ciudad de México, 06720, México; Servicio de Endocrinología, Instituto Nacional de Pediatría, Ciudad de México, 04530, México

Description: This file contains additional information that complements the data and analysis presented in the manuscript. It includes tables and figures that allow a better understanding of the results of the work.

Table S1. Summary of articles with a score of 75% or more.

Reference	Setting	Target molecule	Study population	Detection methods and assays	Written informed consent	Relevant results	Quality (%)
Tomita [1]	United States	IAPP	10 cases of type 1 diabetic pancreata and 8 non-diabetic controls	Immuocytochemical staining	Not applied	Type 1 diabetic islets were generally smaller than control islets and exhibited weaker positivity for both insulin and IAPP. Both normal and type 1 diabetic islets displayed sickle-shaped cytoplasm that was densely positive for insulin and IAPP, without a granular appearance, indicating degenerating insulin cells.	75
Paulsson et al. [2]	United States	IAPP	224 newly diagnosed DM1 children and adolescents	ELISA, Radioimmunoassay, Fluorescent immunoassay, Radioligand binding assay	Yes	11% of the participants (25 out of 224) had concentrations of IAPP exceeding 100 pmol/L. The	100

						increase in IAPP levels did not correlate with C-peptide levels. The elevated plasma IAPP levels in a subset of young individuals with newly diagnosed DM1may increase the risk for IAPP misfolding and the formation of cell toxic amyloid, suggesting a potential pathological role of IAPP aggregation in DM1.	
Westermark et al. [3]	United States	IAPP	Four deceased recipients of islet transplants who had DM1.	Congo red staining	Not applied	The study demonstrated the occurrence of amyloid deposits in islets trans- planted into the liver in three of four patients with DM1.	87.5
Altamirano-Bustamante et al. [4]	México	hIAPP	15 patients (5 with DM1, 5 with DM2, and 5 with obesity) compared to 5 healthy children.	WB, TEM, BCA assay, Thioflavin T fluorescence, SEC, CD Spectroscopy	Yes	The study identifies hexamer oligomers of hIAPP as potential biomarkers for early beta-cell failure. Soluble hIAPP oligomers were detected in serum samples. Different oligomerization states of hIAPP were identified.	87.5
Altamirano-Bustamante et al. [5]	México	Anti-hIAPP cytotoxic oligomers	60 patients with DM1, 32 with DM2, 37 people with obesity and 16 healthy	ELISA, WB, TEM, Cell viability	Yes	RIAO circulate in the blood and can be measured by ELISA; RIAO level increases as the number of complications rises; RIAOs > 3.35 μg/ml is a predic- tor of	100

			adolescents			changes in the current indicators of β-cell damage	
Panagiotopoulos et al. [8]	United States	preproIAPP 5-13	18 HLA-A*0201 patients with DM1, 9 healthy nondiabetic control subjects	Flow cytometry, MHC stabilization assay, Mass spectroscopy, ELISpot	Yes	pre- proIAPP 5-13 is a novel HLA class I epitope recognized by a significant proportion of cytotoxic T-cells from HLA-A*0201 patients with recent-onset DM1 and may prove to be a useful tool for the prediction and/or prevention of this disease.	87.5
Ouyang et al. [9]	United States	IAPP9-17, IGRP215-223, IGRP152-160, IA-2, IA-2(172-180), IA-2(482-490)	24 HLA-A*0201 recent-onset DM1 patients and 11 nondiabetic control subjects	Flow cytometry, Binding, Mass spectroscopy, ELISpot, Liquid chromatography	Yes	Peptides IAPP9-17, IGRP215-223, IGRP152-160, islet IA-2(172-180), and IA-2(482-490) were identified as novel HLA-A*0201–restricted T-cell epitopes in type 1 diabetic patients; it was observed a strong inverse correlation between the binding affinity of -cell peptides to HLA-A*0201 and CTL responses against those peptides in recent-onset type 1 diabetic patients; self-reactive CTLs with specificity for an insulin peptide are frequently present in healthy individuals	75

Beery et al. [6]	United States	IAPP	Patients with DM1 and non-diabetic control donors	H&E, IHC, Congo Red, Image Analysis	Yes	Islet amyloidosis was detected in all three DM1 donors but not in the matches control donors, A scattered distribution of amyloid-positive islets was found in two of the donors, while one donor showed lobular clustering of amyloid-containing islets, primarily in the pancreas tail	87.5
Bruggeman et al. [7]	Switzerland	IAPP	131 DM1 and 111 control organ donors	H&E, ICC	Not applied	Islet amyloid and acute pancreatitis were more common in alcohol users. Acute pancreatitis, chronic pancreatitis and chronic exocrine changes were more common in DM1 donors. Alcohol and/or cocaine use in DM1 organ donors increases exocrine pancreas pathology and islet amyloid deposition.	87.5
S. Zhang et al. [10]	New Zealand	hA	FVB/N mice transgenic for hA cDNA, male mice, mice genotyped together with their respective non-transgenic siblings and mice scarified	BCA assay, ELISA, Immunofluorescence staining, RT-qPCR, Fluorescence microscopy, Light and image microscopy	Not applied	hA- evoked diabetes is associated with age, insulin resistance, progressive islet dysfunction, and cell apoptosis; The various levels of hA elevation cause different extents of oligomer formation in the disease stages, thus eliciting	87.5

			at different stages of diabetes			early- or adult-onset diabetes syndromes, reminiscent of DM1 and DM2, respectively	
Baker et al. [11]	United States	KS20 peptide	NOD and NOD.scid breeding mice	Competitive Peptide Binding assay, Flow cytometry	Not applied	The results suggest that IAPP (and the KS20 peptide) may trigger a broad autoimmune response in NOD mice, as evidenced by the presence of diverse T cell clones reacting to KS20 across different diabetic mice.	87.5
Aida et al. [12]	United States	Beta cells	12 islet autoantibody-positive DM1 patients and 19 age-matched subjects with no diabetes	IHC, immunofluorescence, immunoperpoxidase staining, laser capture microdissection, mass spectroscopy, radioimmunoassay	Yes	Insulitis was observes in al DM1 cases, MHC class I hyperexpression on residual beta cells was a distinctive feature, Exocrine pancreatic inflammation was prominent, No amylin-positive deposition was detected	100
G. Tetz et al. [24]	United Kingdom	Amyloid-producing E.coli	10 children who exhibited autoantibodies (six who developed serum autoantibodies with no progression to DM1, and four who were seroconverted and developed DM1) and eight non-seroconver	CR depletion assay, Double agar overlay	Not applied	The study proposes that amyloid-producing E. coli, their phages, and bacteria-derived amyloid might be involved in activating pro-diabetic pathways in children at risk for DM1.	75

			ted control individuals				
Courtade et al. [27]	United States	IAPP	DM1 patients, DM2 patients, islet transplant recipients, healthy controls and kidney transplant recipients	ELISA, chemiluminescence, DPP-IV degradation assay	Yes	Children with DM1 exhibited significantly reduced plasma concentrations of mature IAPP compared to healthy age-matched controls. The ratio of proIAPP1-48 relative to total IAPP was significantly higher in patients with DM1 (0.613%) compared to healthy controls (0.176%). In adults with DM1, a similar trend was observed, with reduced levels of mature IAPP and proIAPP1-48 relative to healthy controls, along with an increased ratio of proIAPP1-48 to total IAPP.	87.5
Landreh et al. [30]	United States	C-peptide	Human recombinant insulin And C-peptide	Mass spectrometry, Microscopy, Peptide aggregation assay	Not applied	C-peptide modulates the lag time for insulin aggregation, leading to a change in the fibril appearance from regular, extended fibrils to rounded clumps of short fibrils. C-peptide interacts with aggregating insulin and becomes incorporated into the aggregates. The effects observed with insulin and C-peptide	75

						interactions are similar to those of C-peptide on islet amyloid polypeptide fibrillation, indicating that C-peptide may have a broader ability to interact with amyloidogenic proteins derived from pancreatic β-cell granules.	
Potter et al. [31]	Canada	IAPP	Cultured human islets and immune-deficient mice	TEM, Thioflavin T assay, Fluorescent microscope, Thioflavin S staining, TUNEL	Not applied	Heparin promoted the fibrillization of human islet amyloid polypeptide (IAPP) and enhanced its toxicity to INS-1 beta cells; Heparin increased amyloid deposition in cultured human islets, but surprisingly decreased islet cell apoptosis	75
Lv et al. [13]	United Kingdom	α-syn and IAPP	Transgenic mouse model (BAC-α-syn-GFP)	WB, chemiluminescence, Confocal microscopy, Immunofluorescence, IHC	Not applied	STZ treatment caused more severe pathological changes in the pancreatic islets and worsened DM1 symptoms in α-synuclein (α-syn)-overexpressing mice compared to wild-type mice at both one month and three months post-injection. STZ-treated α-syn mice exhibited increased accumulation of α-synuclein and elevated Serine 129 (S129) phosphorylation levels, suggesting enhanced	75

						pathological aggregation.	
Fuchs et al. [17]	United Kingdom	Murine pre-proinsulin 2, IGRP, zinc transporter 8, Ia-2, Ia-2β, glutamic acid decarboxylase 67 (Gad67), chromogranin A, insulinoma amyloid polypeptide, homeobox protein Nkx-2.2	RIP-murine CD80 transgenic mice	ELISPot, Laser scanning confocal microscope	Not applied	RIP- CD80GP mice are a versatile model of antigen specific autoimmune diabetes and may complement existing mouse models of autoimmune diabetes for evaluating CD8+ T cell-targeted prevention strategies.	75
Zhang et al. [33]	United States	hA, FasL	Cultured rat and human insulinoma cells and insolated murine islets	RT-PCR, WB analysis, Immunofluorescence staining, Circular dichroism spectroscopy, Thioflavin T fluorescence assay, Caspase activity assay, Cell death detection enzyme-linked immunosorbent assay	Not applied	hA treatment stimulated Fas and FADD expression in beta-cells; Both blocking antibodies suppressed hA-evoked apoptosis but did not modify its aggregation ;hA-evoked -cell apoptosis was suppressed and rescued by Kp7–6 ;	87.5
Huang et al. [21]	United States	IAPP	HIP and wild-type rats, 7 lean nondiabetic, 12 obese nondiabetic, 14 obese type 2 diabetic, and 8 DM1 human pancreata,	WB, TUNEL assay, chemiluminescence, CHOP staining, IHC, Fluorescent and confocal microscopy,	Not applied	IAPP induces -cell apoptosis by ER stress in INS-1 cells and HIP rats	75

[a]hIAPP: human IAPP; [b]DM1: Diabetes Mellitus Type 1; [c]DM2: Diabetes Mellitus Type 2; [d]ELISA: enzyme-linked immunosorbent assay; [e]TEM: Transmission Electron Microscopy; [f]RIAO: real IAPP oligomers; [g]IAPP: islet amyloid polypeptide; [h]HIP: Human IAPP; [i]IHC: immunohistochemistry; [j]TUNEL: Terminal deoxynucleotidyl transferase-mediated dUTP nick-end-labeling; [k]CHOP: C/EBP homologous protein; [l]ER: Endoplasmic reticulum; [m]INS-1: rat insulinoma; [n]MHC: major histocompatibility complex; [o]INS-1: rat insulinoma; [p]HPLC: rat insulinoma; [q]ELISpot: Enzyme-linked immunospot; [r]IGRP: islet-specific glucose-6-phosphatase catalytic subunit-related protein; [s]RIP-CD80GP: RIP-CD80 x RIP-LCMV-GP; [t]CTLs: cytotoxic T-lymphocytes; [u]hA: human amylin; [v]BCA: Bicinchoninic Acid; [w]RT-qPCR: reverse transcription quantitative polymerase chain reaction; [x]FasL: Fas ligand; [y]FADD: Fas-associated death domain; [z]H&E: hematoxylin and

eosin; [aa]MHC: major histocompatibility complex; [bb]NOD: nondiabetic; [cc]DPP-IV: Dipeptidyl peptidase IV; [dd]STZ: Streptozotocin; [ee]WB: Western Blot.

Table S2. Demonstration of Oligomerization of IAPP in DM1 Pathogenesis and Characteristics of how DM1 was induced.

Author	Settings	Experimental approach	Target population	Characteristics of DM1 (how was induced)	Demonstration of Oligomers in DM1
Tomita [1]	United States	Human subjects research	Pancreatic tissues from DM1 and non-diabetic controls	Not applied	Observations of sickle-shaped cytoplasms in degeneration process were densely stained for IAPP.
Paulsson et al. [2]	United States	Human subjects research	Recent on-set DM1 and healthy children	Not applied	Elevated IAPP concentrations exceeding 100 pmol/L were detected in 11% of the tested DM1 patients
Westermark et al. [3]	United States	Human subjects research	Deceased islet-bearing recipients	Not applied	It was demonstrated the occurrence of amyloid deposits in islets transplanted into the liver in the three of four patients with DM1
Altamirano-Bustamante et al. [4]	México	Human subjects research	Healthy, with clinical data of insulin resistance and DM1 children and adolescents	Not applied	TEM showed the presence of small, medium and large oligomers. In many PTS there are observed small, medium and large amyloid fibers building a network. RIAO aggregated quickly.
Altamirano-Bustamante et al. [5]	México	Human subjects research	Pediatric patients with obesity or diabetes mellitus	Not applied	WB using specific antibodies to detect IAPP oligomers observed different aggregation states in the serum samples from DM1 patients. TEM was used to observe the morphology of

					oligomers and fibers in the serum. Finally, ELISA was used to quantify the RIAO; patients had elevated levels of these oligomers. In DM1 it was seen the effect of very high toxicity of RIAO
Panagiotopoulos et al. [8]	United States	Human subjects research	Individuals with DM1, specifically focusing on HLA-A*0201 patients	Not applied	ELISpot assays showed that PBMCs from recent-onset patients had a notable immune response to the preproIAPP (5-13) peptide. A higher number of reactive cytotoxic T cells was observed compared to long-standing patients and healthy controls. Six of the nine recent-onset patients showed significant immune responses, while none of the long-standing patients showed such responses
Ouyang et al. [9]	United States	Human subjects research	People with recent-onset DM1 who express the HLA-A*0201 allele	Not Applied	-
Beery et al. [6]	United States	Human subjects research	Patients with DM1 and non-diabetic control donors	Not Applied	The study revealed islet amyloidosis. All islets had beta cells displaced towards the periphery of the islet or vascular channels to varying degrees due to intra-islet amyloid deposits. Congo Red staining showed a wide range of numbers of

					amyloid-positive islets per section.. All three donors had insulitis, but insulitic islets did no show amyloidosis.
Bruggeman et al. [7]	Switzerland	Human subjects research	DM1and no-diabetes control organ donors	Not applied	The study shows that islet amyloid deposition, which could include IAPP oligomers or aggregates, was more frequent in alcohol users with DM1.
S. Zhang et al. [10]	New Zealand	Animal study	hA-transgenic mice	Using transgenic FVB/N mice that expressed human amylin (hA) almost exclusively in their pancreatic β-cells	Immunoreactive signals corresponding to AOLIM were observed
Baker et al. [11]	United States	Animal study	NOD and NOD.scid breeding and NOD.IAPP-/- mice	NOD.IAPP-/- mice were created by backcrossing C57BL6.IAPP-/- mice onto the NOD background. Mice were considered diabetic when their blood glucose levels exceeded 15 mmol/l on two consecutive readings	-
Aida et al. [12]	United States	Human subjects research	Islet autoantibody-positive DM1 patients and age-matched subjects with no diabetes	Not applied	-
G. Tetz et al. [24]	United Kingdom	Human subjects research	HLA- matched infants followed from birth until 3 years of age	Not applied	-
Courtade et al. [27]	United States	Human subjects research	Subjects with DM1, DM2, recipients of islets transplants, and healthy controls	Not applied	Individuals with DM1 tend to have higher levels of proIAPP1-48 relative to total IAPP, indicating

					defects in the conversion of IAPP precursors to mature IAPP, possibly contributing to amyloid formation.
Landreh et al. [30]	United States	Cellular research	Human recombinant insulin and C-peptide	Not applied	-
Potter et al. [31]	Canada	Animal and cellular research	Patients Undergoing Islet Transplantation, Human Islets, INS-1 Beta Cells, DM2 Models	Streptozotocin	-
Lv et al. [13]	United Kingdom	Animal study	Transgenic mouse line (BAC-α-syn-GFP) overexpressing human α-syn	Streptozotocin	-
Fuchs et al. [17]	United Kingdom	Animal study	RIP-CD80x RIP-LCMV-GP (RIP-CD80GP) mice	Mice were genetically engineered by crossing RIP-murine CD80 (B7·1) transgenic mice with RIP-LCMV-GP mice. Mice were vaccinated with DNA plasmids encoding pancreatic islet antigens as well as LCMV peptides. Mice were also injected with insulin emulsified in incomplete Freund's adjuvant	-
Zhang et al. [33]	United States	Cellular research	Isolated Mouse Islets, Insulinoma Cell Lines, DM2 Models	Not Applied	-
Huang et al. [21]	United States	Animal and cellular research	HIP and wild-type rats, patients with DM1 and healthy individuals, obese type 2 diabetic, obese nondiabetic, lean nondiabetic human pancreata	Human and rat IAPP precursor protein was clones into an adenovirus vector. INS-1 cells were transducted with the adenovirus constructs to express either	-

				human or rat IAPP to EGFP. HIP rats were used, where human IAPP was overexpressed under the rat insulin gene promoter	

[a]IAPP: islet amyloid polypeptide; [b]DM1: Diabetes Mellitus Type 1; [c]ELISA: enzyme-linked immunosorbent assay; [d]TEM: Transmission Electron Microscopy; [e]RIAO: real IAPP oligomers; [f]HIP: Human IAPP; [g]INS-1: rat insulinoma; [h]PBMCs: Peripheral Blood Mononuclear Cells; [i]AOLIM: amylin oligomer-like immunoreactive material; [j]PTS: pretreated samples; [k]WB: Western Blot.

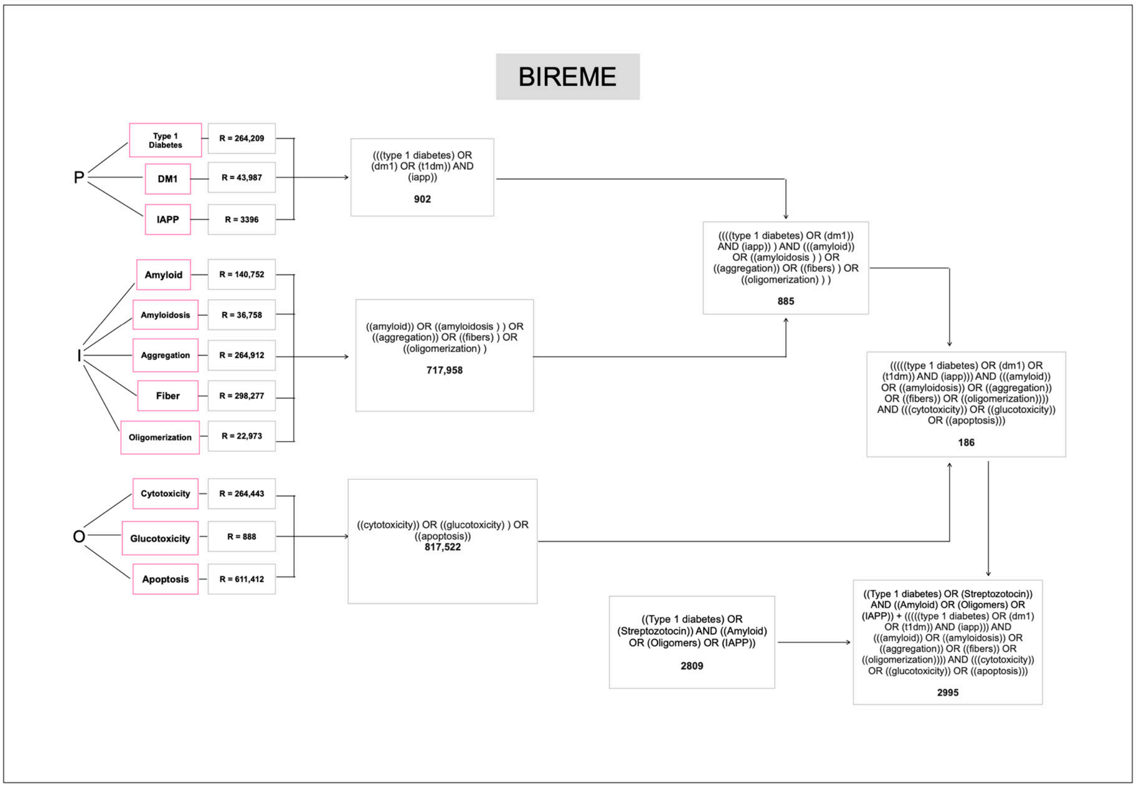

Figure S1. Modified PICO (PIO) approach for the systematic review from the database BIREME. P (Participants), I (Intervention) and O (Outcome).

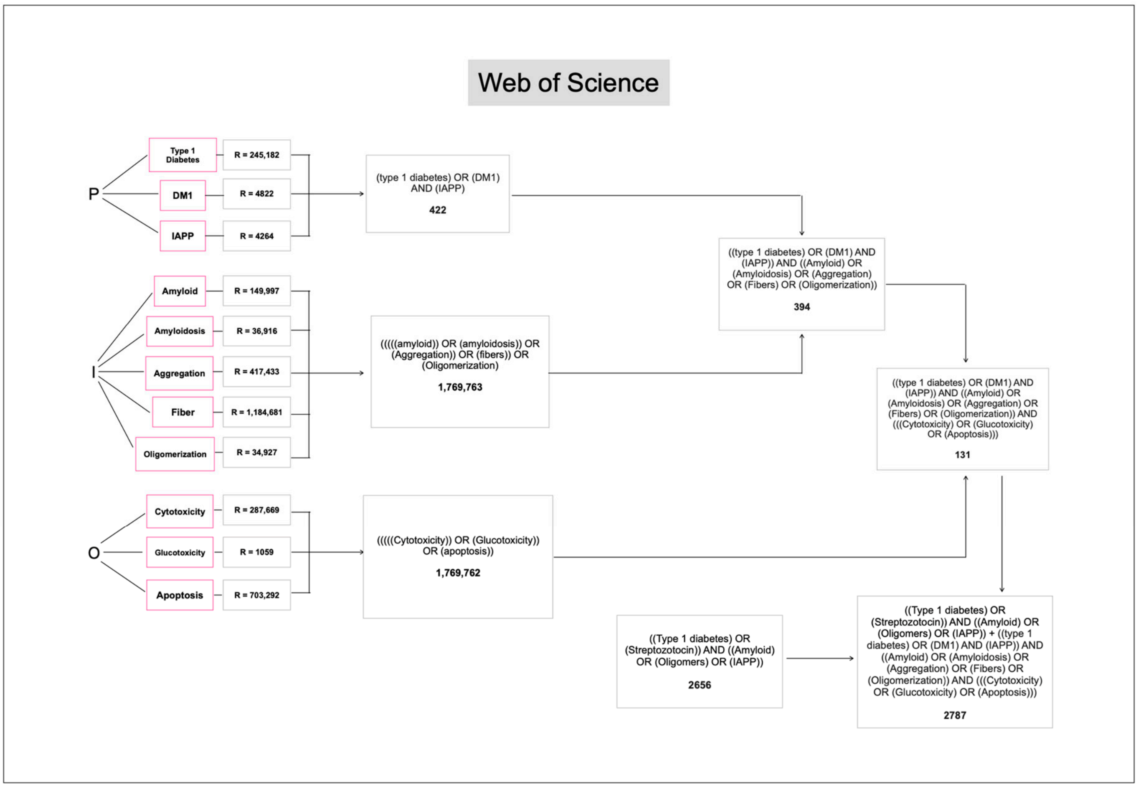

Figure S2. Modified PICO (PIO) approach for the systematic review from the database Web of Science. P (Participants), I (Intervention) and O (Outcome).
